# Olfactory identification, cognition, depressive symptoms, and 5-year mortality in patients with subjective cognitive decline, mild cognitive impairment, and Alzheimer’s disease

**DOI:** 10.1007/s10354-023-01008-6

**Published:** 2023-03-14

**Authors:** Gisela Pusswald, Blaz Dapić, Carina Bum, Eva Schernhammer, Elisabeth Stögmann, Johann Lehrner

**Affiliations:** 1 Department of Neurology, Medical University of Vienna, Vienna, Austria; 2 Department of Epidemiology, Center for Public Health, Medical University of Vienna, Vienna, Austria; 3 Department of Psychiatry and Psychotherapy, Medical University of Vienna, Vienna, Austria

**Keywords:** Olfactory impairment, Cognitive decline, Mortality, Dementia, Aging

## Abstract

**Objective:**

An association between odor and cognitive impairment has been shown in many studies. The objective of the present hospital-based, single-center retrospective study was to assess the impact of odor impairment on the mortality of patients with Alzheimer’s disease (AD), subjective cognitive decline (SCD), and mild cognitive impairment (MCI).

**Methods:**

Odor function was measured by Sniffin Sticks (Burghart Messtechnik, Holm, Germany) and the assessment of self-reported olfactory functioning and olfaction-related quality of life (ASOF) test. Cognitive performance was assessed by an extensive neuropsychological test battery, symptoms of depression were diagnosed with the Geriatric Depressive Scale (GDS). The influence of demographic factors such as gender, age, and education were examined.

**Results:**

Although the univariate analyses and pairwise post hoc comparison showed significant differences for some of the olfactory performance tests/subtests, the multivariate models showed no association between olfactory test performance and mortality among patients with cognitive impairment. “Attention,” a domain of the Neuropsychological Test Battery Vienna (NTBV), as well as depressive symptoms, gender, and age, showed a significant influence on the mortality of the patient group.

**Conclusion:**

Lower olfactory performance showed no impact on mortality. However, decreased cognitive function of “Attention” can be considered as an influential predictor for mortality.

## Introduction

The sense of smell has several important roles. For example, it influences emotional states including enthusiasm, attention, and sexual behavior. It enables the control of food hygiene by warning of rotten food after a person has memorized associations with unpleasant odors, thus protecting against diseases [1]. Presbyosmia, or gradual degeneration of the sense of smell, is a common symptom of aging [2, 3]. More than half of all individuals aged 80 or above are affected by it [4] due to accumulative environmental effects on the olfactory nerve [2], the only cranial nerve exposed to external surroundings [5]. In the elderly population, olfactory impairment is associated with increased mortality [5–11]. One example of this correlation is weight loss [12–14], which can lead to an increased death rate in the geriatric population [15, 16].

The association between odor impairment and cognitive impairment is a well-established fact [17], with Waldton et al. (1974) being the first to connect functional damage of mostly olfactory and gustatory nerves in patients with dementia, emerging at the beginning of the disease with a tendency to progress [18]. Since then, this connection has been very well documented in various epidemiological, neuroimaging, and autopsy studies [17]. Odor impairment has also been associated with other neurodegenerative diseases such as Parkinson’s [19, 20]. The association between neurodegenerative diseases and odor impairment might be explained by the accumulation of pathological proteins in the olfactory epithelium, the olfactory bulb, the entorhinal cortex, and the hippocampus [17]. Furthermore, there are many age-related changes in the olfactory system. For instance, apart from the replacement of the olfactory epithelia with respiratory epithelia, there is a decline in the size and number of patent foramina of the cribriform plate and a considerable decrease in olfactory bulb volume [17]. Furthermore, several longitudinal studies have investigated the conversion rate from mild cognitive impairment (MCI) to Alzheimer’s disease (AD) in association with olfactory impairment. Because olfactory function is linked to changes in neuroanatomical structures, as mentioned above, impairment of such function can be used as an early marker for MCI [21].

The major objective of the present retrospective study was identification of the impact of odor impairment on the 5‑year mortality of patients with cognitive impairment, specifically subjective cognitive decline (SCD), MCI, and AD. The influence of demographic factors such as age, gender, and education; depressive symptoms; cognitive performance; and the cognitive diagnosis itself was also examined. We hypothesized that odor impairment, as well as age, gender, education, depressive symptoms, cognitive performance, and cognitive diagnosis have an impact on the mortality of patients affected by cognitive decline. As there has been little research concerning odor impairment and mortality in cognitively impaired patients, we hoped to gain more knowledge concerning the correlation with the aid of this retrospective study.

## Methods

### Study design

**Fig. 1:**
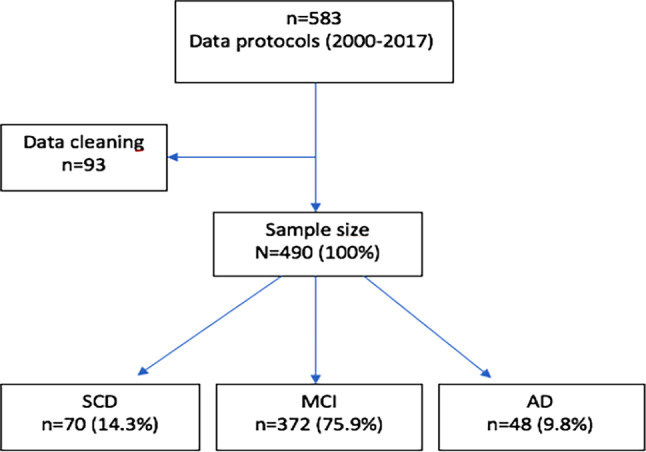
Flowchart on the structure of the patient collective taking into account the exclusion criteria with frequencies and corresponding proportion values regarding the three diagnostic subgroups (*N* = 490)

This study used a retrospective, single-center approach. Test results of 583 cognitively impaired patients from the observation period between January 1998 and December 2017 were available. The present study was based on 490 patient protocols. Those with missing relevant data (e.g., olfactory function) and one patient tested after the defined end of the study (December 31, 2017) were excluded from the subsequent analyses, as shown in Fig. 1. Obvious input errors (e.g., values out of range) in the database were eliminated. Neuropsychological test values were audited for correctness and plausibility.

### Participants

The current study includes patients of both genders above the age of 50 who were treated in the memory outpatient clinic of the Medical University of Vienna.

The study protocol has been approved by the Ethical Committee of the Medical University of Vienna (EK 174/2008) and written informed patient consent to perform this study has been received.

All patients underwent a full physical and neurological examination with use of various screening tests. Furthermore, computed tomography (CT) and magnetic resonance imaging (MRI) scans were performed in most of the patients. The classification into SCD required the presence of subjective memory deterioration as manifested by the seeking of medical help for memory problems and by the concurrent absence of any objectively, measurable cognitive deficits (mean z‑score of each domain greater than −1.5 standard deviation [SD]; Jessen et al., 2014). MCI was determined by a mean z‑score of at least one domain of cognitive functions being below −1.5 SD [22, 27]. AD patients were diagnosed using the NINCDS-ADRDA (National Institute of Neurological Disorders and Stroke-Alzheimer’s Disease and Related Disorders Association).

Inclusion criteria for the study were age over 50 at the time of cognitive and olfactory performance testing, and a cognitive impairment, either self-described or diagnosed by a physician, had to be present.

Patients were excluded from the study if any of the following conditions applied: younger than 50 years; evidence of stroke, as determined by neuroradiological and clinical examination; a history of severe head injury or current psychiatric diagnoses, according to the International Classification of Diseases (ICD)-10 (Dilling et al., 2000), although patients with sub-depressive symptoms were included because sub-depressive symptoms often occur in elderly patients; or any medical condition associated with severe cognitive deterioration, including renal, respiratory, cardiac, and hepatic disease.

Relevant sinonasal diseases (i.e., chronic rhinosinusitis, nasal polyps, etc.) were not evaluated and thus not excluded.

Sociodemographic data are shown in Table 1.

**Table 1:** Sociodemographic data of the subgroups (SCD, MI, AD)

	SCD *n* = 70	MCI *n* = 372	AD *n* = 48
Female age (years)	66 ± 10.5	67.8 ± 9.0	73.9 ± 6.8
Male age (years)	67.9 + 9.6	68.6 + 8.6	70.8 + 8.9
Education (years)	12.4 ± 3.6	12.4 ± 4.1	11.2 ± 3.7
MMSE (0–30)	28.83 ± 1.22	27.84 ± 1.54	24.58 ± 2.23
GDS (0–15)	3.42 ± 3.17	3.72 ± 3.16	4.55 ± 3.5

*MMSE* Mini Mental State Examination, *GDS* Geriatric Depression Scale, *SCD* Subjective Cognitive Decline, *MCI* Mild Cognitive Impairment, *AD* Alzheimer’s Disease

### Neuropsychological instruments

#### Mini-Mental State Exam

The Mini-Mental State Exam (MMSE) is a 30-point questionnaire and a standard tool for cognitive assessment in the clinical setting. It includes items for the assessment of orientation, registration, memory, attention, computation, language, and visual constructive tasks to determine whether cognitive impairment is present or not [23–25]. For the purpose of the study, MMSE was used as a screening for the inclusion of patients, but no further analyses using MMSE were done.

#### Neuropsychological Test Battery Vienna 15

The Neuropsychological Test Battery Vienna 15 (NTBV-15 short) contains various subtests that can be divided into the domains of attention, executive functions, language, and memory. The age concentration test (*Alters-Konzentrations-Test*) and the symbol-counting task from the cerebral insufficiency (CI) test were used to measure attention, as were the TMT A and the planning maze test from the *Nürnberger Alters Inventar* test battery. Furthermore, the interference test from the CI and the phonematic verbal fluency (PWT; letter f) test were also used to evaluate executive functioning. Language was examined using the Boston Naming Test (BNT) and the semantic verbal fluency test (SWT; animals). The verbal selective reminding test (VSRT) was used to evaluate domain memory, with subtests for immediate recall, total recall, delayed recall, and recognition [26–28]. The NTBV can be obtained from www.psimistri.com.

### Olfactory function testing

#### Sniffin’ Sticks©

The 16-item Sniffin’ Sticks (Burghart Messtechnik, Holm, Germany) identification test is an objective test of nasal chemosensory performance based on odor-delivery devices similar to felt-tip pens. These pen-like devices have a length of 14 cm, with an inner diameter of 1.3 cm. The sticks are filled with liquid odorants firstly dissolved in propylene glycol to a total volume of 4 ml. The standardized test procedure involves placing the respective odors in the felt-tip pens for 3 s approximately 2 cm below both nostrils of a patient’s nose. Scores are between 0 and 16 points. The following cut-offs were used: normosmia, identification score 11–16; hyposmia + anosmia, identification score 0–10 [29, 51–53].

#### ASOF

The assessment of self-reported olfactory functioning and olfaction-related quality of life (ASOF), a 12-item questionnaire, was used. This tool can be subdivided into three domains: the one-item, subjective olfactory capability (SOC) scale, the five-item self-reported capability of perceiving specific odors (SRP) scale, and the six-item olfaction-related quality of life (ORQ) scale. Patients were therefore considered to have abnormal olfactory abilities if their SOC scores were ≤ 2.9, their SRP scores were ≤ 2.8 or below, and their ORQ scores were ≤ 3.6.

### Depressive symptoms

#### Geriatric depression scale—short form

The geriatric depression scale, GDS, is a screening instrument for assessing depression specifically in the elderly. The original GDS consists of 30 questions, all of which can be answered with a simple “yes” or “no.” The present study used a shorter form of GDS that comprised 15 questions. The score ranges from 0 to 15, with higher scores indicating depression. Scores 0–4 are suggested to be interpreted as normal, 5–9 as mild depression, and 10–15 as moderate to severe depression [30].

### Statistical analysis

The descriptive and inferential statistical analyses were performed using IBM SPSS® 22 for Mac OSX statistics software (IBM. Corp., Armonk, NY, USA). In inference statistics, the significance level was set at α = 5%, corresponding to the type 1 error. According to this approach, results with *p* ≤ 0.05 were considered significant.

For the description of the characteristic values of metric parameters, the mean value (M), the standard deviation (SD), the span based on minimum (min) and maximum (max), and, for obliquely distributed variables, the alternative measure median (Md) and the associated interquartile range (IQR) were determined and presented. Boxplots were created to illustrate the distribution of metric parameters. To characterize the categories of nominally scaled variables, the frequencies (*n*) and the proportional values (%) were calculated and displayed in bar charts, if applicable. In order to be able to specify the range of the expected value for proportional values, the corresponding 95% confidence interval (CI; lower limit, upper limit) was also specified with the expression. For the 5% error probability of the confidence interval, the corresponding two-tailed *z*-value of 1.96 was used.

In the context of the conclusive statistics, the Mann–Whitney U test was used to compare ordinally scaled measurements between two independent sample groups. This rank-sum test behaves robustly to skewed data, and was preferred to the Student’s *t*-test in this case. Additionally, the non-parametric Kruskal–Wallis test was used as an alternative to one-way ANOVA to perform comparisons of more than two groups.

For further survival analysis, Kaplan–Meier (KM) plots were drawn and survival curves were compared by log-rank tests.

The Cox proportional hazards model (Cox logistic regression) was used to evaluate the influence of certain factors, particularly odor impairment, on patient mortality.

Further on, the present study used an explorative factor analysis (principal component analysis, PCA) to calculate a dimensional reduction of the NTBV test performance. Subsequent orthogonal rotation using the varimax criterion according to Kaiser was performed to structure and gain an overview of the structure of NTBV subtests. The Kaiser–Meyer–Olkin (KMO) coefficient was used to examine whether the extent of the intercorrelations was sufficient for conducting a PCA analysis.

## Results

### Olfactory test performance

**Fig. 2:**
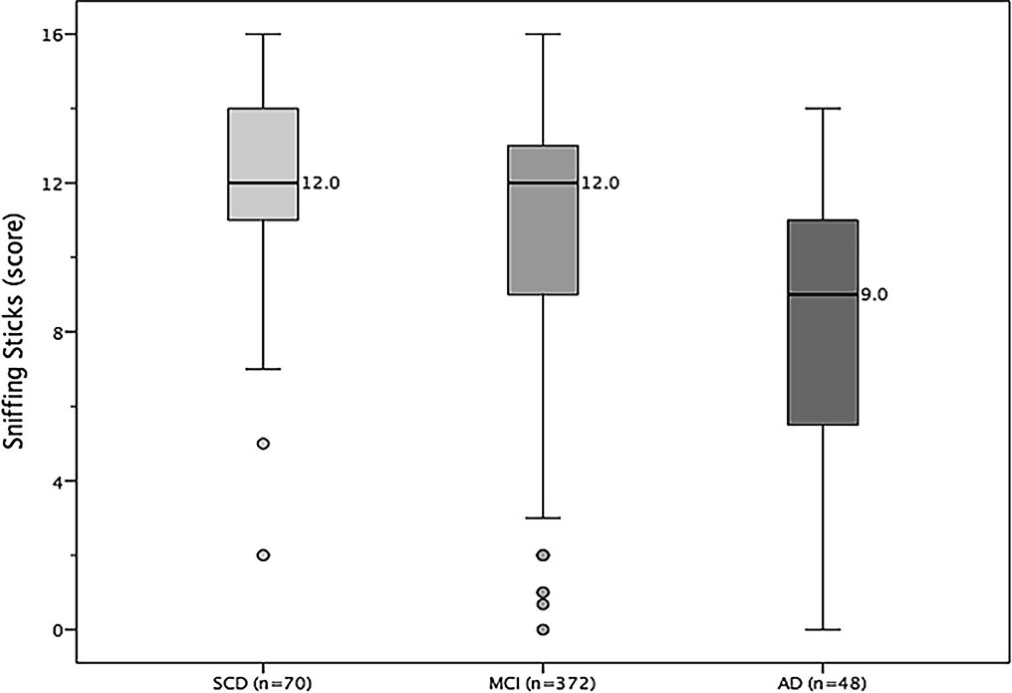
Distribution of olfactory performance (Sniffin’ Sticks identification score, with median) for the diagnostic subgroups. *SCD* Subjective Cognitive Decline, *MCI* Mild Cognitive Disorder, *AD* Alzheimer’s Disease

The performance of olfactory test procedures in the three diagnostic subgroups is shown in Fig. 2. The descriptive key values of olfactory performance regarding diagnostic subgroups are presented in Table 2.

**Table 2:** Characteristic values of olfactory performance testing for the diagnostic subgroups (*n* = cases representing complete data protocols)

Olfactory performance (score range)	SCD	MCI	AD	Total
Identification score (0–16)	*n* = 70	*n* = 372	*n* = 48	*N* = 490
*M* ± *SD*	11.94 ± 2.78	11.17 ± 3.12	8.29 ± 3.38	11.00 ± 3.24
*Md* (IQR)	12.0 (11.0; 14.0)	12.0 (9.0; 13.0)	9.0 (5.3; 11.0)	12.0 (9.0; 13.0)
SOC (0–10)	*n* = 69	*n* = 350	*n* = 36	*n* = 455
*M* ± *SD*	7.29 ± 2.53	7.09 ± 2.53	5.56 ± 3.55	7.00 ± 2.65
*Md* (IQR)	8.0 (5.0; 10.0)	8.0 (5.0; 10.0)	6.0 (3.0; 8.8)	8.0 (5.0; 10.0)
SRP (1–5)	*n* = 70	*n* = 357	*n* = 36	*n* = 463
*M* ± *SD*	4.36 ± 0.87	4.19 ± 1.02	3.91 ± 1.12	4.20 ± 1.01
*Md *(IQR)	4.8 (4.0; 5.0)	4.6 (3.7; 5.0)	4.0 (3.3; 5.0)	4.6 (3.8; 5.0)
ORQ (1–5)	*n* = 70	*n* = 355	*n* = 35	*n* = 460
*M* ± *SD*	4.72 ± 0.51	4.61 ± 0.65	3.89 ± 1.18	4.57 ± 0.71
*Md *(IQR)	5.0 (4.7; 5.0)	5.0 (4.3; 5.0)	4.2 (3.0; 5.0)	5.0 (4.3; 5.0)

**Fig. 3:**
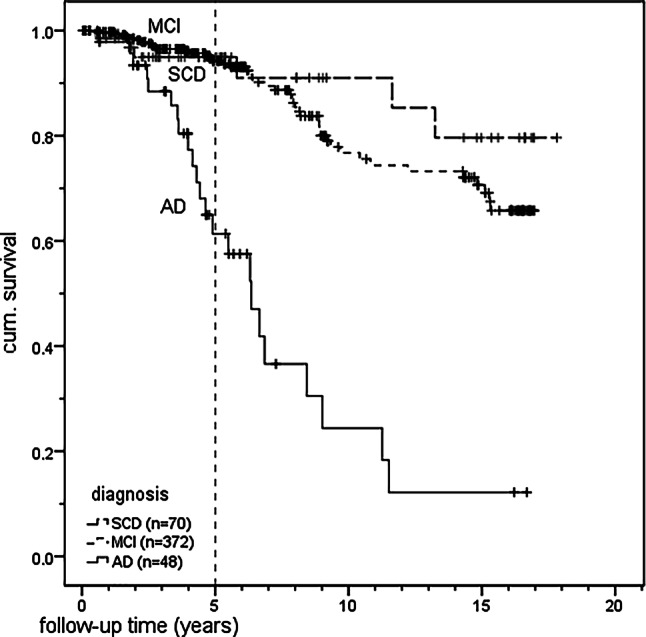
Kaplan–Meier functions for 5‑year survival probability after neuropsychological testing for deceased and censored cases (*n* = 490)

The results showed significant differences for Sniffin’ Sticks identification score, with χ^2^(2) = 39.02, *p* < 0.001; for SOC, with χ^2^(2) = 6.36, *p* = 0.042; and for ORQ, with χ^2^(2) = 18.00, *p* < 0.001. Pairwise post hoc comparisons between the diagnostic subgroups were performed by U‑testing, considering the Bonferroni correction (α* = 0.0167). For Sniffin’ Sticks identification score comparing SCD vs. AD, *p* < 0.001, and for MCI vs. AD, *p* < 0.001, suggesting lower olfactory performance among AD patients. On the other hand, SCD vs. MCI did not differ significantly (*p* = 0.064). The pairwise comparisons between the diagnostic subgroups in SOC did not show any significant differences after considering the Bonferroni correction. Again, for OEQ comparing SCD vs. AD, *p* < 0.001, and MCI vs. AD, *p* < 0.001, suggesting lower olfactory performance among AD patients. On the other hand, SCD vs. MCI did not differ significantly (*p* = 0.185). In contrast, no significant differences between the diagnostic subgroups were found for SRP, with χ^2^(2) = 4.60, *p* = 0.100. Figure 3 illustrates the characteristics of olfactory performance (Sniffin’ Sticks identification score) in a comparison of the diagnostic subgroups.

### NTBV test performance

Table 3 contains specific values and test statistics for the diagnostic subgroups considering NTBV-15 neuropsychological performance.

**Table 3:** Characteristic values of NTBV performance for the diagnostic subgroups (*n* =cases representing complete data protocols)

NTBV subtest	Diagnostic subgroup			Total
	SCD	MCI	AD	
AKT time, concentration I	*n* = 70	*n* = 372	*n* = 48	*N* = 490
*M* ± *SD*	28.89 ± 6.90	36.54 ± 13.39	52.96 ± 20.62	37.06 ± 14.77
*Md* (IQR)	28.0 (24.0; 31.7)	34.0 (27.0; 43.0)	46.5 (39.25; 66.5)	33.0 (27.0; 43.0)
AKT total/time (GZE), concentration II	*n* = 70	*n* = 372	*n* = 48	*n* = 490
*M* ± *SD*	1.98 ± 0.47	1.64 ± 0.53	1.12 ± 0.41	1.63 ± 0.55
*Md* (IQR)	1.96 (1.72; 2.22)	1.58 (1.26; 1.99)	1.13 (0.81; 1.34)	1.61 (1.23; 2.0)
Symbols counting (c.I.)	*n* = 70	*n* = 371	*n* = 47	*n* = 489
*M* ± *SD*	18.30 ± 4.40	22.35 ± 7.31	30.45 ± 10.12	22.56 ± 7.84
*Md *(IQR)	18.0 (15.0; 21.0)	21.0 (17.0; 25.0)	29.0 (21.0; 36.0)	21.0 (17.0; 26.0)
Psychomotor processing speed (TMT A)	*n* = 70	*n* = 372	*n* = 48	*n* = 490
*M* ± *SD*	33.59 ± 10.78	44.61 ± 20.09	75.73 ± 28.74	46.08 ± 22.63
*Md *(IQR)	32.0 (27.0; 40.0)	40.0 (32.0; 53.0)	68.0 (57.0; 97.25)	40.0 (32.0; 55.0)
SWT animals	*n* = 70	*n* = 372	*n* = 48	*n* = 490
*M* ± *SD*	24.96 ± 5.07	21.09 ± 6.30	14.44 ± 4.52	20.99 ± 6.49
*Md *(IQR)	25.0 (21.0; 28.0)	20.0 (16.0; 24.0)	14.0 (12.0; 17.0)	20.0 (16.0; 25.0)
PWT f‑words	*n* = 70	*n* = 371	*n* = 48	*n* = 489
*M* ± *SD*	12.57 ± 3.67	9.93 ± 3.97	6.73 ± 3.15	9.99 ± 4.10
*Md *(IQR)	13.00 (10.0; 15.0)	10.00 (7.0; 13.0)	6.0 (5.0; 9.0)	10.0 (7.0; 13.0)
Naming (BNT)	*n* = 70	*n* = 372	*n* = 48	*n* = 490
*M* ± *SD*	14.51 ± 0.76	13.86 ± 1.31	12.42 ± 2.09	13.81 ± 1.44
*Md *(IQR)	15.0 (14.0; 15.0)	14.0 (13.0; 15.0)	13.0 (11.0; 14.0)	14.0 (13.0; 15.0)
VSRT total	*n* = 70	*n* = 372	*n* = 48	*n* = 490
*M* ± *SD*	8.40 ± 2.03	7.35 ± 2.07	4.69 ± 1.68	7.24 ± 2.23
*Md *(IQR)	8.5 (7.0; 10.0)	7.0 (6.0; 9.0)	4.5 (3.25; 6.0)	7.0 (6.0; 9.0)
VSRT imm. recall	*n* = 70	*n* = 372	*n* = 48	*n* = 490
*M* ± *SD*	53.97 ± 9.01	46.15 ± 11.18	28.54 ± 7.12	45.55 ± 12.25
*Md *(IQR)	55.0 (47.5; 61.0)	46.0 (38.0; 54.0)	29.0(23.25; 32.75)	46.0 (37.0; 55.0)
VSRT del. recall	*n* = 70	*n* = 372	*n* = 48	*n* = 489
*M* ± *SD*	11.49 ± 2.54	9.20 ± 3.41	3.58 ± 2.14	8.98 ± 3.74
*Md *(IQR)	12.0 (9.0; 13.25)	9.0 (7.0; 12.0)	3.5 (2.0; 5.0)	9.0 (6.0; 12.0)
VSRT recognition	*n* = 70	*n* = 372	*n* = 47	*n* = 486
*M* ± *SD*	14.59 ± 0.65	13.68 ± 2.12	9.86 ± 3.38	13.45 ± 2.46
*Md *(IQR)	15.0 (14.38; 15.0)	14.5 (13.5; 15.0)	10.5 (7.5; 12.5)	14.5 (13.0; 15.0)
Maze time	*n* = 70	*n* = 372	*n* = 47	*n* = 489
*M* ± *SD*	30.49 ± 10.20	44.52 ± 22.43	63.04 ± 28.12	44.29 ± 23.1
*Md *(IQR)	29.0 (23.0; 36.25)	39.0 (30.0; 52.0)	57.0 (42.0; 76.0)	39.0 (29.0; 52.0)
Maze total/time	*n* = 70	*n* = 372	*n* = 47	*n* = 489
*M* ± *SD*	0.55 ± 0.17	0.41 ± 0.18	0.28 ± 0.14	0.42 ± 0.19
*Md *(IQR)	0.54 (0.41; 0.66)	0.39 (0.27; 0.52)	0.26 (0.17; 0.35)	0.39 (0.28; 0.53)
Interference time (c.I.)	*n* = 70	*n* = 372	*n* = 47	*n* = 489
*M* ± *SD*	20.17 ± 4.62	24.34 ± 6.89	34.04 ± 31.0	24.67 ± 7.85
*Md *(IQR)	19.00 (17.0; 23.0)	23.0 (20.0; 27.0)	31.0 (25.0; 41.0)	23.0 (19.0; 28.0)
Interference total/time (c.I.)	*n* = 70	*n* = 371	*n* = 36	*n* = 477
*M* ± *SD*	1.76 ± 0.39	1.48 ± 0.38	1.09 ± 1.1	1.49 ± 0.41
*Md *(IQR)	1.79 (1.48; 2.0)	1.47 (1.22; 1.7)	1.1 (0.74; 1.43)	1.48 (1.22; 1.74)

Test results revealed, as expected, that all NTBV subtest performances in all three diagnostic subgroups differed significantly (*p*-values < 0.001), indicating a hierarchical order: SCD > MCI > AD.

### Dimensional reduction of NTBV subtests

In order to provide a summary of the NTBV subtests in terms of dimensional reduction, a PCA with subsequent varimax orthogonal rotation according to Kaiser was performed, as shown in Table 4.

**Table 4:** Factor loadings of NTBV-15 (short version) subtests regarding four dimensions (*n* = 476, listwise exclusion)

NTBV-15 (short version) subtest	Domain				Communality
	1	2	3	4	h_i_^2^
Interference time (c.I.)	***−0.855***	−0.171	−0.111	−0.207	0.82
AKT time, concentration I	***−0.825***	0.197	0.115	0.213	0.76
Interference total/time (c.I.)	***0.805***	−0.125	−0.306	−0.058	0.78
AKT total/time (GZE), concentration II	***0.778***	0.161	0.318	0.102	0.74
Symbols counting (c.I.)	***−0.647***	−0.079	−0.249	−0.220	0.54
Naming (BNT)	***0.309***	0.150	0.163	0.285	0.23
VSRT delayed recall	*0.181*	**0.876**	0.176	0.117	0.85
VSRT learning performance	*0.211*	**0.861**	0.177	0.207	0.86
VSRT recognition	0.106	***0.778***	−0.015	−0.038	0.62
VSRT word span	0.130	***0.759***	0.175	0.240	0.68
Maze time	−0.301	*−0.140*	**−0.875**	−0.109	0.89
Maze total/time	0.306	*0.142*	**0.843**	0.155	0.85
Psychomotor processing speed (TMT A)	*−0.508*	−0.273	**−0.522**	−0.115	0.62
PWT‑f correct	0.159	0.096	0.034	***0.869***	0.79
Animals correct (SWT), word fluency	0.333	0.252	0.266	***0.641***	0.66
Eigenvalue (λ)	3.86	3.02	2.22	1.56	Σ 10.66
Proportion of explained variance	25.8%	20.1%	14.8%	10.4%	71.1%

Communality h_i_^2^ explains variance percentage, representing the sum of squares of squared factor loadings of each row of an NTBV-15 (short version) subtest. Eigenvalue λ, on the other hand, represents the sum of squares of each factor’s column and comprises values > 1. Numbers in bold correspond to the same factor loadings

The amount of explained variance reached 71.1% assuming four domains, representing the cognitive structure. Regarding the loading of the NTBV-15 (short version) subtests in each of the factors, the domains may be designated as following:

1. *attention,*
2. *verbal memory,*
3. *executive function,*
4. *language.*

In order to compare the diagnostic subgroups with regard to the heterogeneity of the variances (*p*-values ≤ 0.047) to the four factor scores (domains), Welch ANOVAs were conducted. Hereby, all domains revealed significant differences (*p*-values < 0.001), with effect sizes ranging from small (*executive functions*) to large (*verbal memory*), as shown in Table 5.

**Table 5:** Characteristics of NTBV-15 (short version) z‑factor scores (µ = 0, σ = 1) performance considering the diagnostic subgroups (total *n* = 476)

NTBV-15 domain	SCD (*n* = 70)	MCI (*n* = 370)	AD (*n* = 36)	*p*-value	Effect η^2^
1 Attention	0.433 ± 0.761	0.003 ± 0.956	−0.874 ± 1.278	< 0.001**	0.09
2 Verbal memory	0.463 ± 0.649	0.047 ± 0.938	−1.381 ± 1.025	< 0.001**	0.18
3 Executive function	0.391 ± 0.715	−0.037 ± 1.008	−0.377 ± 1.181	< 0.001**	0.03
4 Language	0.488 ± 0.796	−0.044 ± 1.000	−0.498 ± 1.014	< 0.001**	0.06

**p ≤ 0.01

Follow-up pairwise comparisons were conducted using the Games–Howell post hoc procedure, taking different sample sizes into account and considering heterogenous variances and Bonferroni adjustment, α* = 0.0167. As a result, test performances for 1) *attention*, 2) *verbal memory,* 3) *executive function*, and 4) *language* revealed the following hierarchy: SCD > MCI > AD; *p-*values ≤ 0.002, except comparisons of *executive function* MCI vs. AD, *p* = 0.231, and *language *MCI vs. AD, *p* = 0.036.

### Life expectancy and mortality

#### Mortality

The survival function, taking censored cases into account, was calculated using the Kaplan–Meier (KM) survival function. The KM method considers information about deceased and censored survivors within the follow-up time. In a further step, Kaplan–Meier functions for survival estimation after neuropsychological testing of the three diagnostic subgroups were calculated, as shown in Fig. 3. This procedure is based on the consideration of follow-up time intervals after the assessment with the neuropsychological test battery.

The specific survival probabilities of the three diagnostic subgroups were derived from this analysis, as shown in Table 6.

**Table 6:** Cumulative 5‑year survival (in percent) of patients

Diagnostic subgroup	N	5‑year survival likelihood (%)
SCD	70	95.0
MCI	372	94.2
AD	48	61.3
Total	490	90.8

The log-rank test, comparing the overall equality of survival distributions, revealed a significant difference between the three diagnostic subgroups, χ^2^(2) = 69.140, *p* < 0.001. Post hoc pairwise comparisons between SCD vs. MCI showed a nonsignificant result, χ^2^(1) = 0.829, *p* = 0.363, whereas SCD vs. AD, χ^2^(1) = 27.388, *p* < 0.001, and MCI vs. AD, χ^2^(1) = 62.220, *p* < 0.001, yielded a significantly poorer survival for AD patients.

Table 7 displays the estimated mean and median survival times in years of the three diagnostic subgroups. It should be noted that no median time was estimated for the SCD and MCI patients because their overall survival estimates were > 50%.

**Table 7:** Estimated means and medians for survival time (years) depending on the three diagnostic subgroups

Diagnostic subgroup	Mean^a^				Median			
	Estimate	SE	95% CI		Estimate	SE	95% CI	
			LL	UL			LL	UL
SCD	15.91	0.74	14.46	17.36	–	–	–	–
MCI	14.23	0.37	13.52	14.95	–	–	–	–
AD	7.41	0.89	5.67	9.15	6.36	0.72	4.94	7.77
Overall	14.16	0.37	13.44	14.88	–	–	–	–

^a^Estimation is limited to the longest survival time if it is censored

#### Life expectancy

This section initially presents the descriptive results regarding survival until study end. Table 8 displays the key values of age at time of death of already deceased patients until the reference date of December 31, 2017, among the three diagnostic groups.

**Table 8:** Frequencies and (row) percentages of deceased patients until reference date; key values of age (years) at death of deceased patients within the sample (*N* = 490)

Diagnosis Subgroup	Deceased	M ± SD	min–max	Md	IQR	Mean rank
SCD (*n* = 70)	6 (8.6%)	82.7 ± 7.8	69.2–90.3	86.1	77.5; 87.0	46.17
MCI (*n* = 372)	45 (12.1%)	78.6 ± 8.4	59.2–91.6	79.5	72.7; 86.5	35.80
AD (*n* = 48)	23 (47.9%)	79.4 ± 8.7	58.2–89.6	80.2	76.8; 86.3	38.57
Total (*N* = 490)	74 (15.1%)	79.2 ± 8.4	58.2–91.6	80.2	73.1; 86.7	–

The result of the corresponding Kruskal–Wallis test showed a nonsignificant difference of age of already deceased participants among the diagnostic subgroups, x^2^(2) = 1.312, *p* = 0.519. Age at death of deceased patients in different impaired groups seemed to be comparable.

#### Prediction of mortality

Using the Cox proportional hazards model and taking follow-up time and censored cases into account, four successive hierarchical blocks of predictors were made using the enter method within each block. In the first approach (model I) olfactory performance (Sniffin’ Sticks identification score and ASOF variables scoring; block 1), demographic characteristics (age, sex, education; block 2), depression (GDS short; block 3), and cognition (NTBV short four-factor scores; block 4) were addressed as predictors for the dichotomous criterion (recorded death within the observation period up to study end, December 31, 2017, vs. survival). A total of 424 complete data protocols could be considered, with 58 (13.7%) deceased cases. Table 9 displays the results of the model testing at the last step, including all four blocks.

**Table 9:** Coefficients of predictors (model I) using Cox proportional hazards model regarding death as a criterion (four blocks, *n* = 424)

Predictor	B	SE	Wald ^2^ (df = 1)	*p*-value	HR	95% CI HR	
						LB	UB
Identification score (0–16)	−0.043	0.048	0.810	0.368	0.958	0.872	1.052
SOC (0–10)	−0.119	0.067	3.220	0.073	0.887	0.779	1.011
SRP (1–5)	0.106	0.152	0.484	0.486	1.112	0.825	1.499
ORQ (1–5)	0.277	0.218	1.611	0.204	1.319	0.860	2.021
Age (years)	0.067	0.020	10.800	0.001**	1.069	1.027	1.112
Sex (male = 0, female = 1)	−0.696	0.300	5.403	0.020*	0.498	0.277	0.897
Education	−0.060	0.041	2.155	0.142	0.942	0.870	1.020
GDS (0–15)	0.103	0.044	5.527	0.019*	1.108	1.017	1.207
*NTBV*							
1 Attention	−0.421	0.132	10.170	0.001**	0.656	0.507	0.850
2 Verbal memory	−0.280	0.169	2.744	0.098	0.756	0.542	1.053
3 Executive function	−0.032	0.151	0.046	0.831	0.968	0.721	1.301
4 Language	−0.169	0.142	1.409	0.235	0.845	0.639	1.116

**p ≤ 0.01, *p ≤ 0.05

The results indicate that olfactory performance, including Sniffin’ Sticks identification score and the ASOF subtests in the first block, had no significant explanatory value for the probability of death (*p*-value ≥ 0.073), considering the follow-up time component. In the second block, gender and education were revealed as risk factors for mortality, (*p*-values ≤ 0.020). *Depressive symptoms* (GDS) in the third block also indicated the relative risk for mortality (*p* = 0.019). Finally, in the fourth block, the cognitive structure, represented by four NTBV factor scores, was examined for an explanatory value on mortality. It could be observed that *attention, *with an HR of 0.5656 and 95% CI [0.507; 0.850], may serve as a significant, protective predictor for a reduced risk of mortality.

## Discussion

The challenge and simultaneous question were whether and to what extent reduced olfactory function in cognitively impaired patients indicates an increased mortality risk. The study examined the explanatory value of olfactory impairment in relation to mortality in three diagnostic subgroups, also considering depressive functions and cognitive structure. The evaluation of olfactory performance in terms of prediction of mortality risk, analyzed with the Cox proportional hazards model, showed that if the diagnostic subgroup, sociodemographic factors, depressive symptoms, and the cognitive structure based on the NTBV-15 factor scores are taken into account, olfactory performance cannot provide any significant explanatory value, even after dichotomized data that distinguish between patients with odor impairment and patients with normal olfactory functions are created.

The nonsignificant association between olfactory impairment and mortality is in contrast to a number of population studies in which results have shown that an adult or elderly individual with odor impairment is at a higher risk of death than a person with normosmia (normal sense of smell) [5–11]. Schubert et al. (2017) correlated olfactory impairment with an increased risk of death among older patients, unlike visual and hearing impairments, after adjustment to atherosclerosis and inflammatory marker levels [10]. Association between objective olfactory impairment and death was found by Choi et al. (2021) among patients aged 65 years or older, even after adjusting for covariates, but not among patients between the ages of 40 and 64 or among those with self-reported (subjective) olfactory dysfunction [7]. One of the ways in which we might be able to explain our findings is through potential cofounders such as age, depression, or gender. All three were significant predictors of mortality in our study’s patient population. Nonetheless, the systematic review and meta-analysis by Pang et al. (2022) found that olfactory impairment remains a significant predictor of mortality even after adjusting for potential cofounders, although a possible cofounder link or covariate association through cognitive decline, weakness, or systemic diseases could not be entirely excluded [5]. Furthermore, Liu et al. (2019) found that dementia, Parkinson’s disease, and weight loss are a common cause of death among older patients with poorer olfactory functions [6]. Contrary to the rest of the mentioned studies, Gopinath et al. (2012) found no association between total mortality and olfactory impairment in elderly patients after adjusting for cognitive impairment [13].

A role of education as a protective factor against mortality has been shown in other studies [31–33], but in our study, no association between education and mortality of patients with cognitive decline was found. Furthermore, gender was found to have a significant influence in the study’s patient population, with female patients having a lower mortality rate than their male counterparts. Female gender has been seen as a protective factor, with a lower mortality rate in comparison to the male gender, in a multitude of studies [34–38]. Leschak et al. (2018) found a correlation between olfactory dysfunction and increased mortality among the adult and older female patient population, but not for the male patient population. Physical closeness was observed to have an influence on the aforementioned correlation among female patients, but other social factors, such as social network size and emotional closeness, did not seem to affect it [39].

Depression and age have been shown to be important mortality predictors in this study. Depression, as well as other mental disorders, has been found to increase the risk of death among affected individuals in comparison to the general population [40]. Going further, major depressive symptoms among patients with mild dementia increase the risk of death 2.5-fold, as shown by Petersen et al. (2017) [41]. Age, as expected, has an influence on the mortality of patients with cognitive decline, as corroborated by Lee et al. (2018) [35].

The results of our PCA factor analysis indicate four dimensions (*attention, verbal memory, executive function, *and *language*) of the NTBV subtests, which can be assumed to contribute to revealing the human cognitive structure. The factors may help to explain the cognitive performance behavior of the participants. Of the four NTBV subtests, only the *attention* domain showed a significant influence on the survival of the patient population. In fact, it was the only factor in our Cox regression model that showed a significant influence on survival. A decreased NTBV *attention *factor score may be regarded as an influencing predictor of mortality. Cognitive performance, as examined by neuropsychological test batteries, has been shown to be a good mortality predictor [42]. Besides *attention*, the* verbal memory *domain showed a tendency to influence the mortality of the patient population. This influence, however, remained nonsignificant.

The severity of cognitive decline was not shown to have an influence on the survival of the patient population in the Cox regression model. This was further substantiated by the Kruskal–Wallis test showing that the age of death among the cognitive groups did not differ from one another. This challenges the notion of most other studies that claim that dementia is an impact factor for mortality among the older population [43–50]. Although contradictory to most, Lee et al.’s (2018) research supports our finding, claiming that age and other comorbidities have a greater influence on mortality than cognitive decline itself [35]. Similarly, a population study from Sweden did not find dementia to attenuate the association between olfactory loss and mortality, suggesting that olfactory loss might mark deteriorating health irrespective of dementia [53], further supporting our findings.

## Limitations

There were several limitations to this study. First, it should be emphasized that the olfactory identification test was carried out once as a status snapshot. Other qualities of olfaction such as threshold sensitivity or discrimination were not measured in this study. Individual variations may play a role.

Previous ear, nose, and throat interventions or injuries as well as smoking may also influence olfactory performance. Furthermore, there was a small sample size restriction concerning neuropsychological testing, as it would have been unethical to perform the entire test battery on patients who are clearly suffering from Alzheimer’s dementia and therefore had poor cognitive performance. It should be noted that the NTBV factor analysis was carried out with those cases that had complete data protocols, which resulted in a loss of 20 (4.1%) out of 490 cases in total. In addition, due to the long period of 17 years of data collection, heterogeneous technical adjustments were made, which was reflected in the completeness of the test data entered. Besides, patient dementia medication status, which could influence the quality of olfactory function, was not collected.

## Conclusion

In conclusion, lower olfactory performance as assessed via Sniffin’ Sticks identification score and the ASOF test as well as by using a multivariate approach could not achieve significant results. Furthermore, decreased NTBV *attention *can be considered as an influential predictor for mortality. In addition, the Cox model showed that the diagnostic subgroup has no explanatory value for mortality. From the results of the present study, only neurocognitive *attention,* depressive symptoms represented by GDS-15, age, and gender may have an important impact on the progression of Alzheimer’s disease in terms of mortality. However, further research is needed to substantiate these results.

## References

[1] Nef P (1998). How we smell: the molecular and cellular bases of olfaction. News Physiol Sci.

[2] Papazian EJ, Pinto JM (2021). Olfactory loss and aging: connections with health and well-being. Chem Senses.

[3] Pinto JM, Wroblewski KE, Kern DW, Schumm LP, McClintock MK (2015). The rate of age-related olfactory decline among the general population of older U.S. Adults. J Gerontol A Biol Sci Med Sci.

[4] Attems J, Walker L, Jellinger KA (2015). Olfaction and aging: a mini-review. Gerontology.

[5] Pang NY, Song H, Tan BKJ (2022). Association of olfactory impairment with all-cause mortality: a systematic review and meta-analysis. JAMA Otolaryngol Head Neck Surg.

[6] Liu B, Luo Z, Pinto JM (2019). Relationship between poor olfaction and mortality among community-dwelling older adults: a cohort study. Ann Intern Med.

[7] Choi JS, Jang SS, Kim J, Hur K, Ference E, Wrobel B (2021). Association between olfactory dysfunction and mortality in US adults. JAMA Otolaryngol Head Neck Surg.

[8] Pinto JM, Wroblewski KE, Kern DW, Schumm LP, McClintock MK (2014). Olfactory dysfunction predicts 5-year mortality in older adults. plos One.

[9] Wilson RS, Yu L, Bennett DA (2011). Odor identification and mortality in old age. Chem Senses.

[10] Schubert CR, Fischer ME, Pinto AA (2017). Sensory impairments and risk of mortality in older adults. J Gerontol A Biol Sci Med Sci.

[11] Siegel JK, Wroblewski KE, McClintock MK, Pinto JM (2019). Olfactory dysfunction persists after smoking cessation and signals increased cardiovascular risk. Int Forum Allergy Rhinol.

[12] Purdy F, Luo Z, Gardiner JC (2020). Olfaction and changes in body composition in a large cohort of older U.S. Adults. J Gerontol A Biol Sci Med Sci.

[13] Gopinath B, Sue CM, Kifley A, Mitchell P (2012). The association between olfactory impairment and total mortality in older adults. J Gerontol A Biol Sci Med Sci.

[14] Boyce JM, Shone GR (2006). Effects of ageing on smell and taste. Postgrad Med J.

[15] Alibhai SM, Greenwood C, Payette H (2005). An approach to the management of unintentional weight loss in elderly people. CMAJ.

[16] Marton KI, Sox HC, Krupp JR (1981). Involuntary weight loss: diagnostic and prognostic significance. Ann Intern Med.

[17] Park SJ, Lee JE, Lee KS, Kim JS (2018). Comparison of odor identification among amnestic and non-amnestic mild cognitive impairment, subjective cognitive decline, and early Alzheimer’s dementia. Neurol Sci.

[18] Waldton S (1974). Clinical observations of impaired cranial nerve function in senile dementia. Acta Psychiatr Scand.

[19] Van Regemorter V, Hummel T, Rosenzweig F, Mouraux A, Rombaux P, Huart C (2020). Mechanisms linking olfactory impairment and risk of mortality. Front Neurosci.

[20] Braak H, Del Tredici K (2017). Neuropathological staging of brain pathology in sporadic parkinson’s disease: separating the wheat from the chaff. J Parkinsons Dis.

[21] Jung HJ, Shin IS, Lee JE (2019). Olfactory function in mild cognitive impairment and Alzheimer’s disease: a meta-analysis. Laryngoscope.

[22] Petersen RC, Smith GE, Waring SC, Ivnik RJ, Kokmen E, Tangelos EG (1997). Aging, memory, and mild cognitive impairment. Int Psychogeriatr.

[23] Cummings JL (1993). Mini-mental state examination. JAMA.

[24] Tatari F, Farnia F, Kazemi F (2011). Mini mental state examination (MMSE) in first episode of psychosis. Iran. J. Psychiatry.

[25] Escobar JI, Burnam A, Karno M, Forsythe A, Landsverk J, Golding JM (1986). Use of the Mini-Mental State Examination (MMSE) in a community population of mixed ethnicity. Cultural and linguistic artifacts. J Nerv Ment Dis.

[26] Lehrner J, Maly J, Gleiss A, Dal-Bianco EAP (2007). The Vienna Neurophysiological Test Battery (VNTB) for detecting Alzheimer’s Dementia: standardization norms, validations. Psychol Osterr.

[27] Pusswald G, Moser D, Gleiß A (2012). Prevalence of mild cognitive impairment subtypes in patients attending a memory outpatient clinic—comparison of two modes of mild cognitive impairment classification. Results of the Vienna Conversion to Dementia Study. Alzheimers Dement.

[28] Rosas AG, Stogmann E, Lehrner J (2021). Individual cognitive changes in subjective cognitive decline, mild cognitive impairment and Alzheimer’s disease using the reliable change index methodology. Wien Klin Wochenschr.

[29] Kobal G, Klimek L, Wolfensberger M (2000). Multicenter investigation of 1036 subjects using a standardized method for the assessment of olfactory function combining tests of odor identification, odor discrimination and olfactory thresholds. Eur Arch Otorhinolaryngol.

[30] Yesavage JA, Brink TL, Rose TL (1982). Development and validation of a geriatric depression screening scale: a preliminary report. J Psychiatr Res.

[31] Montez JK, Hummer RA, Hayward MD, Woo H, Rogers RG (2011). Trends in the educational gradient of U.S. adult mortality from 1986 to 2006 by race, gender, and age group. Res Aging.

[32] Agüero-Torres H, Fratiglioni L, Guo Z, Viitanen M, Winblad B (1999). Mortality from dementia in advanced age. J Clin Epidemiol.

[33] Rogers RG, Hummer RA, Everett BG (2013). Educational differentials in US adult mortality: an examination of mediating factors. Soc Sci Res.

[34] Mitchell SL, Kiely DK, Hamel MB, Park PS, Morris JN, Fries BE (2004). Estimating prognosis for nursing home residents with advanced dementia. JAMA.

[35] Lee KC, Hsu WH, Chou PH, Yiin JJ, Muo CH, Lin YP (2018). Estimating the survival of elderly patients diagnosed with dementia in Taiwan: a longitudinal study. plos One.

[36] Heyman A, Peterson B, Fillenbaum G, Pieper C (1996). The consortium to establish a registry for Alzheimer’s disease (CERAD). Part XIV: Demographic and clinical predictors of survival in patients with Alzheimer’s disease. Neurology.

[37] Lapane KL, Gambassi G, Landi F, Sgadari A, Mor V, Bernabei R (2001). Gender differences in predictors of mortality in nursing home residents with AD. Neurology.

[38] Connors MH, Ames D, Boundy K (2016). Predictors of mortality in dementia: the PRIME study. J Alzheimers Dis.

[39] Leschak CJ, Eisenberger NI (2018). The role of social relationships in the link between olfactory dysfunction and mortality. plos One.

[40] Walker ER, McGee RE, Druss BG (2015). Mortality in mental disorders and global disease burden implications: a systematic review and meta-analysis. JAMA Psychiatry.

[41] Petersen JD, Waldorff FB, Siersma VD, Phung TKT, Bebe A, Waldemar G (2017). Major depressive symptoms increase 3-year mortality rate in patients with mild dementia. Int J Alzheimers Dis.

[42] Weatherbee SR, Allaire JC (2008). Everyday cognition and mortality: performance differences and predictive utility of the Everyday Cognition Battery. Psychol Aging.

[43] Heun R, Schoepf D, Potluri R, Natalwala A (2013). Alzheimer’s disease and co-morbidity: increased prevalence and possible risk factors of excess mortality in a naturalistic 7-year follow-up. Eur Psychiatry.

[44] Bassuk SS, Wypij D, Berkman LF (2000). Cognitive impairment and mortality in the community-dwelling elderly. Am J Epidemiol.

[45] Kawas CH, Brookmeyer R (2001). Aging and the public health effects of dementia. n Engl J Med.

[46] Wolfson C, Wolfson DB, Asgharian M (2001). A reevaluation of the duration of survival after the onset of dementia. n Engl J Med.

[47] Gambassi GFL, Lapane KL, Sgadari A, Mor V, Bernabei R, the SAGE Study Group (1999). Predictors of mortality in patients with Alzheimer’s disease living in nursing homes. J Neurol Neurosurg Psychiatry.

[48] James BD, Leurgans SE, Hebert LE, Scherr PA, Yaffe K, Bennett DA (2014). Contribution of Alzheimer disease to mortality in the United States. Neurology.

[49] Hane FT, Robinson M, Lee BY, Bai O, Leonenko Z, Albert MS (2017). Recent progress in Alzheimer’s disease research, part 3: diagnosis and treatment. J Alzheimers Dis.

[50] Ganguli MM, Dodge HH, Shen C, Pandav RS, DeKoskoy ST (2005). Alzheimer disease and mortality A 15-year epidemiological study. Arch Neurol.

[51] Hummel T, Kobal G, Gudzil H, Mackay-Sim A (2007). Normative data for the “Sniffin’ Sticks” including tests of odor identification, odor discrimination, and olfactory thresholds: An upgrade based on a group of more then 3000 subjects. Eur Arch Otorhinolaryngol.

[52] Oleszkiewicz A, Schriever V, Hähner A, Hummel T (2019). Updated Sniffin’ Sticks normative data based on an extended sample of 9139 subjects. Eur Arch Otorhinolaryngol.

[53] Ekström I, Sjölund S, Nordin S, Nordin Adolfsson A, Adolfsson R, Nilsson LG, Larsson M, Olofsson JK (2017). Smell Loss Predicts Mortality Risk Regardless of Dementia Conversion. J Am Geriatr Soc.

